# Intermittent Ibandronate Maintains Bone Mass, Bone Structure, and Biomechanical Strength of Trabecular and Cortical Bone After Discontinuation of Parathyroid Hormone Treatment in Ovariectomized Rats

**DOI:** 10.1007/s00223-017-0255-6

**Published:** 2017-02-28

**Authors:** Satoshi Takeda, Sadaoki Sakai, Keisuke Tanaka, Haruna Tomizawa, Kenichi Serizawa, Kenji Yogo, Koji Urayama, Junko Hashimoto, Koichi Endo, Yoshihiro Matsumoto

**Affiliations:** 1 Fuji Gotemba Research Laboratories, Product Research Department, Chugai Pharmaceutical Co., Ltd, 1-135 Komakado, Gotemba, Shizuoka 412-8513 Japan; 2 Product Marketing and Management Department, Taisho Toyama Pharmaceutical Co., Ltd, 3-25-1 Takada, Toshima-ku, Tokyo 170-8635 Japan; 3 Primary Lifecycle Management Department, Chugai Pharmaceutical Co., Ltd, 2-1-1 Nihombashi Muromachi, Chuo-ku, Tokyo 103-8324 Japan; 4 Medical Science Department, Chugai Pharmaceutical Co., Ltd, 2-1-1 Nihombashi Muromachi, Chuo-ku, Tokyo 103-8324 Japan

**Keywords:** Bone biomechanical strength, Bone mineral density, Histomorphometry, Ibandronate, Parathyroid hormone

## Abstract

Although parathyroid hormone (PTH) expresses an anabolic effect on bone mass, the increased bone mass disappears once PTH treatment is withdrawn. Therefore, sequential treatment with anti-bone-resorptive agents is required to maintain bone mass after PTH treatment. We examined the effect of sequential treatment with ibandronate (IBN), a nitrogen-containing bisphosphonate, following PTH in ovariectomized (OVX) rats. Wistar-Imamichi rats (27 weeks old) were ovariectomized and treated with PTH (10 µg/kg, s.c.; 5 times/week; PTH group) for 8 weeks from 8 weeks after OVX. Thereafter, PTH was withdrawn and rats were administered IBN (10 µg/kg, s.c.; every 4 weeks; PTH-IBN group) or vehicle (PTH-Veh group) for another 8 weeks. PTH increased bone mineral density (BMD) measured by dual-energy X-ray absorptiometry and biomechanical strength in the lumbar spine and femur as compared to the disease control rats. BMD and biomechanical strength in the PTH-Veh group were lower than in the PTH group, whereas in the PTH-IBN group they were maintained at the level of the PTH group. Microstructure of the trabecular and cortical bone in the PTH-IBN group was not significantly different from that in the PTH group. In histomorphometric analysis of the lumbar vertebra, eroded surface and osteoclast surface in the PTH-Veh group were no different from those in the PTH group, whereas they were lower in the PTH-IBN group. Osteoid surface, osteoblast surface, and mineralize surface decreased in both PTH-IBN and PTH-Veh groups compared to the PTH group, and these parameters in the PTH-IBN group were lower than in the PTH-Veh group. These results indicated that intermittent IBN after PTH treatment suppressed bone turnover and maintained BMD, biomechanical strength, and microstructure in the lumbar spine and femur of OVX rats.

## Introduction

Agents for the treatment of osteoporosis fall into two major classes: anti-bone-resorptive agents and anabolic agents. There are several types of anti-resorptive treatment, such as bisphosphonates, receptor activator of nuclear factor kappa-B ligand inhibitors, selective estrogen receptor modulators, and vitamin D analogues. On the other hand, human parathyroid hormone (PTH)—either PTH(1–34) or full-length human PTH(1–84)—is the only anabolic agent currently approved for treatment of osteoporosis [1].

PTH increases osteoblast numbers and bone formation via stimulation of osteoblastogenesis and inhibition of osteoblast apoptosis [2]. In postmenopausal women with prevalent vertebral fractures, PTH is reported to increase bone mineral density (BMD) of the spine and reduce the risk of vertebral fractures [3]. Favorable effects of PTH on BMD and fracture risk are also reported in men with osteoporosis [4]. Owing to its high potential to increase BMD and reduce fracture risk, PTH is used for the treatment of osteoporosis patients that have a high risk of fracture. PTH also improves the microstructure of trabecular bone and the collagen cross-link ratio in bone [5, 6]. However, because carcinogenicity studies in rats have found that PTH induces osteosarcomas, the use of PTH is limited to no more than 2 years [7].

Although PTH treatment increases lumbar spinal BMD, the increased BMD declines after PTH is withdrawn [8, 9]. Therefore, administration of anti-bone-resorptive agents is recommended after discontinuation of PTH treatment [4, 10–12]. Ibandronate (IBN), a nitrogen-containing bisphosphonate, is widely used in the treatment of osteoporosis. It can be administered at extended dosing intervals in both intravenous and oral formulations [13, 14]. Monthly intravenous administration of IBN significantly increased BMD with a reduction in bone turnover markers and reduced the incidence of vertebral fractures in patients with primary osteoporosis [14]. Concurrent and sequential therapies of PTH(1–84) with IBN similarly increased the BMD of the lumbar spine and hip over 2 years [15]. However, how IBN affects bone after PTH treatment are not yet clear. In the current study, we examined the effect of sequential treatment with IBN following PTH in ovariectomized rats.

## Materials and Methods

### Experimental Design

**Fig. 1:**
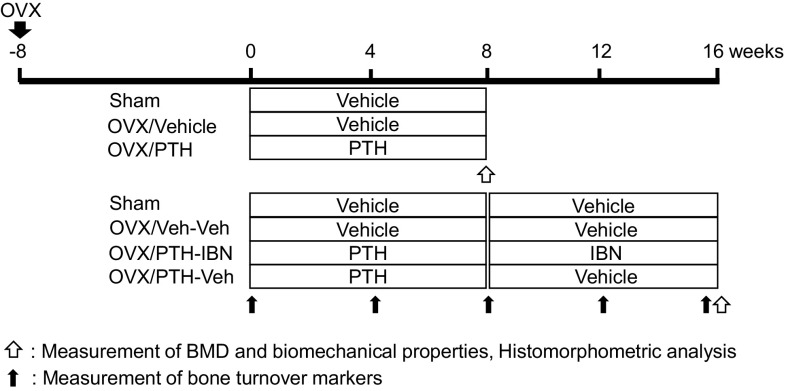
Experimental design. Eight weeks after ovariectomy (OVX), rats were treated with PTH(1–34) (10 µg/kg, s.c., five times/week) or vehicle for 8 weeks. Thereafter, PTH treatment was withdrawn and the PTH-treated rats were administered ibandronate for another 8 weeks (10 µg/kg, s.c., every 4 weeks; IBN) or vehicle. Bone mineral density (BMD) and biomechanical properties were measured, and bone histomorphometry was performed at 8 and 16 weeks. Bone turnover markers were measured every 4 weeks. *n* = 10 for each group

Twenty-five-week-old female Wistar-Imamichi rats were purchased from the Institute for Animal Reproduction (Ibaraki, Japan) and were acclimatized for 2 weeks under standard laboratory conditions at 20–26 °C, 30–70% humidity, and a 12 h:12 h light/dark cycle. All animals had free access to tap water and standard commercial rodent chow (CE-2; CLEA Japan, Inc., Tokyo, Japan). Animals were ovariectomized (OVX) or sham-operated (Sham) at 27 weeks of age, and were left untreated for 8 weeks. Rats were then randomized according to their BMD and body weight into seven groups (Fig. 1). Three of the OVX groups received subcutaneous injections of human PTH(1–34) (10 µg/kg per day, five times per week; Peptide Institute, Inc., Osaka, Japan), and the remaining OVX groups and Sham groups received subcutaneous injections of vehicle (phosphate–citrate-buffered solution consisting of 16.7 mM disodium hydrogenphosphate, 8.4 mM citric acid, 100 mM sodium chloride, and 0.05% Tween-80; pH 5.0; 1 mL/kg per day) [16]. After 8 weeks of treatment, rats in three groups (Sham, OVX/Vehicle, and OVX/PTH) were euthanized, and treatment in the remaining groups was continued for an additional 8 weeks: PTH-treated groups were switched to subcutaneous injections of IBN (10 µg/kg, once every 4 weeks; Chugai Pharmaceutical Co., Ltd., Tokyo, Japan) or vehicle (saline), and vehicle-treated OVX and Sham groups continued to receive vehicle. The dosages of PTH and IBN were determined by reference to previous reports as doses sufficient to increase BMD and bone biomechanical strength in OVX rats [17, 18]. Tetracycline (20 mg/kg) and calcein (6 mg/kg) were injected subcutaneously for bone labeling at 8 and 3 days, respectively, prior to necropsy.

Blood and urine were collected every 4 weeks. Blood was collected from the jugular vein under isoflurane anesthesia and centrifuged to obtain serum. Urine was collected using individual metabolic cages. Serum and urine were stored at −40 °C until biochemical analysis.

At the end of the treatment, animals were euthanized by exsanguination from the abdominal aorta under isoflurane anesthesia. The lumbar spine and bilateral femurs were excised. The second through fourth lumbar vertebrae (L2–L4) and the right femur were retained in 70% ethanol. The fifth lumbar vertebra (L5) and the left femur were wrapped in saline-soaked gauze and stored at −30 °C prior to biomechanical testing. This study was performed according to the experimental protocol approved by the Institutional Animal Care and Use Committee at Chugai Pharmaceutical Co., Ltd.

### Measurement of BMD

For measurement of BMD for grouping of animals, animals were anesthetized using isoflurane, and the BMD of L2–L5 was measured in vivo by dual-energy X-ray absorptiometry with a DCS-600EX bone densitometer (pixel size: 1 mm × 0.5 mm, Aloka Co., Ltd., Tokyo, Japan). For measurement of the BMD in excised bone samples, the BMD of the lumbar spine (L2–L4) and of the right femur was determined using a DCS-600EX bone densitometer. For data analysis, the femur was divided into ten equal segments along its major axis and BMD was calculated in 3 parts: proximal femur (the mean BMD of the 3 proximal segments), middle femur (the mean BMD of the 4 middle segments), and distal femur (the mean BMD of the 3 distal segments).

### Biomechanical Testing

Biomechanical strength was performed with a mechanical testing machine (TK-252C; Muromachi Kikai Co., Ltd., Tokyo, Japan). For the femur 3-point bending test, the upper loading device was aligned at the expected breaking point of the femoral shaft on the anterior side. The span between the two lower supports was set at 15 mm. The load was applied at a rate of 20 mm/min. Prior to the compression test of the L5 vertebral body, the vertebral arch and end plates were cut off with a diamond saw to obtain a specimen with planoparallel ends. The loading rate of the compression test was set at 2.5 mm/min. Maximum load and work to failure were determined from the load–displacement curve, and ultimate stress and toughness were calculated [19]. Stiffness was determined from the slope of the linear elastic region of the load–displacement curve, and Young’s modulus was calculated. Moment of inertia of the femoral shaft was measured at the expected breaking point by peripheral quantitative computed tomography (pQCT; XCT Research M; Stratec Medizintechnik, Pforzheim, Germany) using Cortmode 1. Cross-sectional area at the mid-section of the L5 vertebra was quantified by pQCT using Contmode 2 and Peelmode 20.

### Bone Histomorphometry

Bone histomorphometry was performed on the L3 vertebra and femoral diaphysis. Specimens were fixed in 70% ethanol and stained with Villanueva bone stain. After dehydration, the specimens were embedded in methyl methacrylate. Five-micrometer-thick sections of the L3 vertebral body were prepared to evaluate trabecular bone. For the femoral diaphysis, 20- to 30-µm-thick cross-cut ground sections were obtained for evaluation of cortical bone. Measurements of static and dynamic parameters were collected with an image analyzing system (WinROOF 2013; Mitani Corp., Tokyo, Japan). The secondary spongiosa (approximately 4.416 mm^2^) located 0.5 mm away from the growth plate and 0.15 mm from the margin of the dorsal cortical shell of the L3 vertebral body was used to assess trabecular bone. The following variables were measured: bone volume (BV/TV, %), trabecular thickness (Tb.Th, µm), trabecular number (Tb.N, /mm), trabecular separation (Tb.Sp, µm), eroded surface (ES/BS, %), osteoclast surface (Oc.S/BS, %), osteoid surface (OS/BS, %), osteoblast surface (Ob.S/BS, %), mineralized surface (MS/BS, %), mineral apposition rate (MAR, µm/day), bone formation rate (BFR/BS, µm^3^/µm^2^/year), tissue area (T.Ar, mm^2^), marrow area (Ma.Ar, mm^2^), cortical area (Ct.Ar, mm^2^), cortical width (Ct.Wi, mm), endocortical perimeter (Ec.Pm, mm), and periosteal perimeter (Ps.Pm, mm). Nomenclature and units used in this study follow the report of the American Society for Bone and Mineral Research Histomorphometry Nomenclature Committee [20].

### Biochemical Analysis

Urinary deoxypyridinoline (DPD) was measured using an Osteolinks-DPD kit (DS Pharma Biomedical, Osaka, Japan) and the data were corrected for urinary creatinine (Cr) concentration. Cr was measured with an autoanalyzer (TBA-120FR; Toshiba Medical Systems Co., Tochigi, Japan). Serum osteocalcin (OCN) was measured using an Osteocalcin Rat ELISA System (GE Healthcare Japan Co., Tokyo, Japan).

### Statistical Analysis

All data are presented as the mean ± SEM. The differences between the groups were analyzed with one-way analysis of variance (ANOVA). Statistical differences between individual groups were evaluated with Tukey’s multiple comparison test or Dunnett’s multiple comparison test. Statistical comparisons between the Sham group and the Vehicle or Veh-Veh groups, and between the Vehicle group and the PTH group were performed by unpaired *t* test. For all tests, *P* < 0.05 was considered statistically significant. Bonferroni correction was applied for analysis of bone turnover markers. Statistical analysis was carried out using JMP (SAS Institute Inc., Cary, NC, USA).

## Results

### BMD

**Fig. 2:**
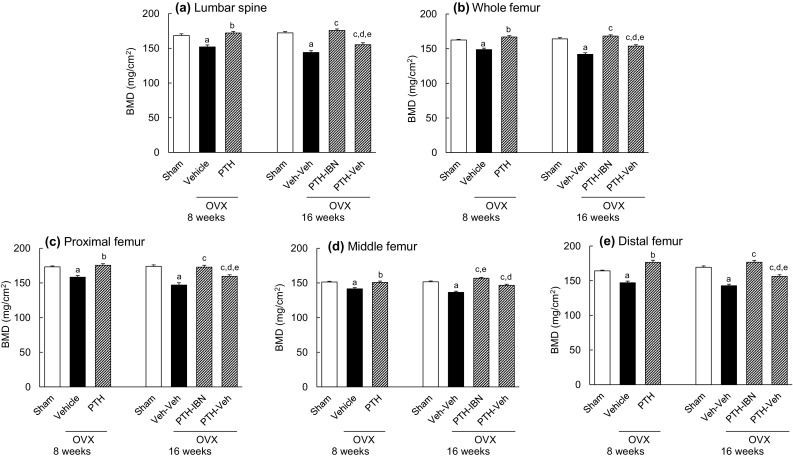
Bone mineral density of the lumbar spine and femur. PTH(1–34) (10 µg/kg, s.c., five times/week) or vehicle was administered to ovariectomized (OVX) rats for 8 weeks from 8 weeks after OVX surgery. Ibandronate (10 µg/kg, s.c., every 4 weeks; IBN) or vehicle was administered sequentially for another 8 weeks following PTH treatment. **a** lumbar spine, **b** whole femur, **c** proximal femur, **d** middle femur, **e** distal femur. Data are presented as mean + SEM. (*n* = 10 for each group). *a P* < 0.05 versus Sham by unpaired *t* test. *b P* < 0.05 versus Vehicle by unpaired *t* test. *c P* < 0.05 versus Veh-Veh; *d P* < 0.05 versus PTH-IBN by Tukey’s multiple comparison test. *e P* < 0.05 PTH versus PTH-IBN or PTH-Veh by Dunnett’s multiple comparison test

OVX induced a significant reduction in the BMD of the lumbar spine and the femur compared to that in the Sham group (Fig. 2a–e). BMD in the PTH group was significantly higher than in the Vehicle group at 8 weeks. At 16 weeks, the BMD of the lumbar spine and the femur in the PTH-IBN and PTH-Veh groups was significantly higher than in the Veh-Veh group, and the BMD in the PTH-IBN group was significantly higher than in the PTH-Veh group. The BMD of the lumbar spine and the proximal, distal, and whole femur in the PTH-Veh group was significantly lower than in the PTH group, whereas the BMD in the PTH-IBN group was not significantly different from that in the PTH group (Fig. 2a–c, e). The BMD of the middle femur in the PTH-IBN group was significantly higher than in the PTH group (Fig. 2d).

### Bone Biomechanical Strength

**Fig. 3:**
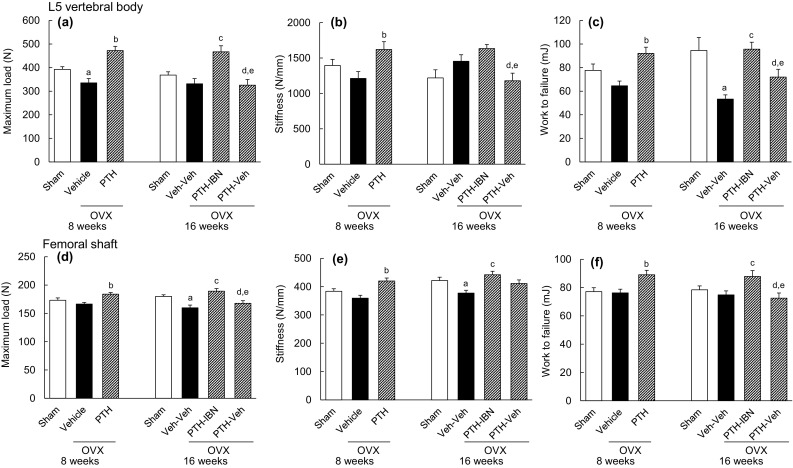
Biomechanical strength of the L5 vertebral body and the femoral shaft. PTH(1–34) (10 µg/kg, s.c., five times/week) or vehicle was administered to ovariectomized (OVX) rats for 8 weeks from 8 weeks after OVX surgery. Ibandronate (10 µg/kg, s.c., every 4 weeks; IBN) or vehicle was administered sequentially for another 8 weeks following PTH treatment. Maximum load, stiffness, and work to failure for the L5 vertebral body (**a, b, c**) and the femoral shaft (**d, e, f**) were determined by compression test and 3-point bending test, respectively. Data are presented as mean + SEM. (*n* = 10 for each group). *a P* < 0.05 versus Sham by unpaired *t* test. *b P* < 0.05 versus Vehicle by unpaired *t* test. *c P* < 0.05 versus Veh-Veh; *d P* < 0.05 versus PTH-IBN by Tukey’s multiple comparison test. *e P* < 0.05 PTH versus PTH-IBN or PTH-Veh by Dunnett’s multiple comparison test

In the compression test of the L5 vertebral body, OVX resulted in significant decreases in maximum load, ultimate stress, and toughness compared to the Sham group at 8 weeks (Fig. 3a; Table 1). In the PTH group, the values of all biomechanical parameters of the L5 vertebral body were higher than in the Vehicle group (Fig. 3a–c; Table 1). At 16 weeks, maximum load, work to failure, ultimate stress, and toughness in the PTH-IBN group were significantly higher than in the Veh-Veh group, and all biomechanical strength parameters of the L5 vertebral body were significantly higher in the PTH-IBN group than in the PTH-Veh group (Fig. 3a–c; Table 1). In the PTH-Veh group, all biomechanical strength parameters were significantly lower than in the PTH group, whereas these parameters in the PTH-IBN group were not significantly different from those in the PTH group.

**Table 1:** Material biomechanical properties of L5 the vertebral body and femoral shaft

	8 weeks			16 weeks			
	Sham	OVX		Sham	OVX		
		Vehicle	PTH		Veh-Veh	PTH-IBN	PTH-Veh
L5 vertebral body							
Ultimate stress (MPa)	40.9 ± 1.2	34.4 ± 1.9^a^	47.5 ± 2.2^b^	38.8 ± 1.5	34.4 ± 1.8	48.3 ± 2.3^c^	32.6 ± 2.4^d, e^
Young’s modulus (MPa)	759.2 ± 48.4	646.1 ± 52.5	845.6 ± 71.3^b^	660.8 ± 63.0	783.8 ± 42.5	881.6 ± 26.0	613.4 ± 55.0^c,d,e^
Toughness (MPa)	1.55 ± 0.10	1.27 ± 0.08^a^	1.79 ± 0.10^b^	1.95 ± 0.24	1.07 ± 0.06^a^	1.90 ± 0.10^c^	1.38 ± 0.12^d,e^
Femoral shaft							
Ultimate stress (MPa)	164.9 ± 6.2	164.3 ± 4.1	171.6 ± 3.3	166.4 ± 4.2	153.1 ± 3.3^a^	171.5 ± 2.9^c^	154.7 ± 2.4^d,e^
Young’s modulus (MPa)	4286.7 ± 140.9	4143.8 ± 139.2	4523.9 ± 201.8	4553.1 ± 151.7	4242.4 ± 177.3	4700.9 ± 228.1	4348.8 ± 141.8
Toughness (MPa)	6.27 ± 0.35	6.46 ± 0.31	7.21 ± 0.20	6.23 ± 0.27	6.14 ± 0.17	6.85 ± 0.30	5.87 ± 0.29^d,e^

Mean ± SEM (*n* = 10 for each group)

^a^ *P* < 0.05 versus Sham by unpaired *t* test

^b^ *P* < 0.05 versus Vehicle by unpaired *t* test

^c^ *P* < 0.05 versus Veh-Veh;

^d^ *P* < 0.05 versus PTH-IBN by Tukey’s multiple comparison test

^e^ *P* < 0.05 PTH versus PTH-IBN or PTH-Veh by Dunnett’s multiple comparison test

*Veh* vehicle, *IBN* ibandronate

In the 3-point bending test of the femoral shaft, PTH treatment resulted in higher maximum load, stiffness, and work to failure than was observed in the Vehicle group at 8 weeks (Fig. 3d–f). OVX induced significant decreases in maximum load, stiffness, and ultimate stress of the femoral shaft in the Veh-Veh group compared to the Sham group at 16 weeks (Fig. 3d, e: Table 1). In the PTH-IBN group, maximum load, stiffness, work to failure, and ultimate stress of the femoral shaft were significantly higher than in the Veh-Veh group, and maximum load, work to failure, ultimate stress, and toughness were significantly higher than in the PTH-Veh group (Fig. 3d–f; Table 1). Maximum load, work to failure, ultimate stress, and toughness in the PTH-Veh group were significantly lower than in the PTH group, whereas in the PTH-IBN group these parameters were maintained at the level of the PTH group (Fig. 3d, f; Table 1).

### Bone Histomorphometry

OVX induced a significant reduction in BV/TV in the trabecular bone of the L3 vertebra, accompanied by significant decreases in Tb.Th and Tb.N, and a significant increase in Tb.Sp compared to the Sham group at 8 weeks (Table 2). PTH resulted in higher BV/TV and Tb.Th and lower Tb.Sp compared to the Vehicle group. In the PTH-IBN and PTH-Veh groups, BV/TV and Tb.N were significantly higher than in the Veh-Veh group, and Tb.Sp was significantly lower than in the Veh-Veh group. Tb.Th was significantly higher in the PTH-IBN group than in the Veh-Veh group. BV/TV and Tb.Th were significantly higher in the PTH-IBN group than in the PTH-Veh group. BV/TV and Tb.Th in the PTH-Veh group were significantly lower than in the PTH group, whereas in the PTH-IBN group they were not significantly different from those in the PTH group.

**Table 2:** Histomorphometry of the trabecular bone of the L3 vertebra

	8 weeks			16 weeks			
	Sham	OVX		Sham	OVX		
		Vehicle	PTH		Veh-Veh	PTH-IBN	PTH-Veh
BV/TV (%)	31.9 ± 1.5	22.9 ± 1.1^a^	32.5 ± 1.4^b^	31.0 ± 1.2	20.1 ± 1.3^a^	35.4 ± 1.7^c^	26.4 ± 1.5^c,d,e^
Tb.Th (μm)	87.0 ± 3.6	76.4 ± 2.9^a^	104.5 ± 4.4^b^	82.9 ± 2.2	74.6 ± 3.6	108.9 ± 4.8^c^	85.3 ± 4.0 ^d,e^
Tb.N (/mm)	3.67 ± 0.10	2.99 ± 0.08^a^	3.12 ± 0.09	3.74 ± 0.10	2.67 ± 0.08^a^	3.25 ± 0.08^c^	3.09 ± 0.09^c^
Tb.Sp (μm)	187.1 ± 7.8	260.2 ± 9.4^a^	218.1 ± 9.0^b^	186.5 ± 7.8	302.2 ± 13.1^a^	200.7 ± 9.0^c^	240.8 ± 10.9^c,d^
ES/BS (%)	8.41 ± 0.72	10.49 ± 1.09	11.35 ± 0.78	6.89 ± 0.44	10.04 ± 0.91^a^	6.41 ± 0.40^c,e^	10.45 ± 0.78^d^
Oc.S/BS (%)	1.51 ± 0.17	2.34 ± 0.38	2.54 ± 0.34	1.72 ± 0.15	2.44 ± 0.29^a^	1.30 ± 0.09^c,e^	2.36 ± 0.24^d^
OS/BS (%)	2.03 ± 0.35	11.10 ± 1.30^a^	16.70 ± 1.38^b^	3.13 ± 0.65	14.64 ± 1.98^a^	0.10 ± 0.05^c,e^	13.21 ± 1.14^d,e^
Ob.S/BS (%)	0.88 ± 0.17	4.75 ± 0.67^a^	8.53 ± 1.10^b^	1.40 ± 0.26	5.37 ± 0.97^a^	0.03 ± 0.02^c,e^	4.62 ± 0.74^d,e^
MS/BS (%)	4.11 ± 0.45	11.66 ± 1.21^a^	22.02 ± 1.19^b^	5.18 ± 0.70	13.19 ± 0.98^a^	2.12 ± 0.21^c,e^	14.43 ± 0.92^d,e^
MAR (μm/day)	1.15 ± 0.05	1.15 ± 0.04	1.43 ± 0.02^b^	1.31 ± 0.04	1.30 ± 0.05	ND	1.33 ± 0.03^f^
BFR/BS (μm^3^/μm^2^/year)	17.2 ± 2.0	48.9 ± 5.3^a^	115.5 ± 7.3^b^	24.5 ± 3.3	63.4 ± 6.2^a^	ND	70.5 ± 5.8^f^

Mean ± SEM (*n* = 10 for each group)

^a^ *P* < 0.05 versus Sham by unpaired t- *t* est

^b^ *P* < 0.05 versus Vehicle by unpaired *t* test

^c^ *P* < 0.05 versus Veh-Veh;

^d^ *P* < 0.05 versus PTH-IBN by Tukey’s multiple comparison test

^e^ *P* < 0.05 PTH versus PTH-IBN or PTH-Veh by Dunnett’s multiple comparison test

^f^ *P* < 0.05 versus PTH by unpaired t test

*Veh* vehicle, *IBN* ibandronate, *ND* not detected

OVX showed a tendency to increase bone resorption parameters (ES/BS and Oc.S/BS) and significantly increased bone formation parameters (OS/BS, Ob.S/BS, MS/BS, and BFR/BS) in the L3 vertebra at 8 weeks as compared to the Sham group (Table 2). PTH did not affect bone resorption parameters but significantly increased bone formation parameters compared to the Vehicle group. Bone resorption parameters in the PTH-IBN group were significantly lower than in the Veh-Veh group or PTH group, whereas in the PTH-Veh group they did not change compared to the Veh-Veh group or PTH group. OS/BS, Ob.S/BS, and MS/BS in the PTH-IBN group were significantly lower than in the Veh-Veh group or PTH group. Bone formation parameters in the PTH-Veh group were not significantly different from those in the Veh-Veh group, but they were significantly lower than in the PTH group. OS/BS, Ob.S/BS, and MS/BS in the PTH-IBN group were significantly lower than in the PTH-Veh group. MAR and BFR/BS in the PTH-IBN group were not able to be determined because double fluorescence bone labeling was not observed.

In the cortical bone of the femoral diaphysis, OVX significantly increased T.Ar, Ma.Ar, and Ec.Pm, and significantly decreased Ct.Wi compared to the Sham group at 8 weeks (Table 3). PTH treatment showed significantly higher Ct.Ar and Ct.Wi, and lower Ma.Ar and Ec.Pm compared to the Vehicle group. OVX and PTH treatment did not change Ps.Pm. In the PTH-IBN group, Ct.Ar and Ct.Wi were significantly higher than in the Veh-Veh group or PTH-Veh group, and Ma.Ar and Ec.Pm were significantly lower than in the Veh-Veh group or PTH-Veh group. In the PTH-Veh group, Ct.Wi was significantly higher than in the Veh-Veh group, and Ma.Ar, Ct.Ar, and Ec.Pm were not significantly different from those in the Veh-Veh group. In the PTH-Veh group, Ma.Ar and Ec.Pm were significantly higher than in the PTH group, and Ct.Ar and Ct.Wi were significantly lower, whereas these structural parameters in the PTH-IBN group were not significantly different from those in the PTH group.

**Table 3:** Histomorphometry of the cortical bone of the femoral diaphysis

	8 weeks			16 weeks			
	Sham	OVX		Sham	OVX		
		Vehicle	PTH		Veh-Veh	PTH-IBN	PTH-Veh
T.Ar (mm^2^)	9.82 ± 0.13	10.20 ± 0.12^a^	10.32 ± 0.16	10.13 ± 0.18	10.26 ± 0.20	10.21 ± 0.18	10.39 ± 0.15
Ma.Ar (mm^2^)	3.77 ± 0.09	4.32 ± 0.08^a^	3.90 ± 0.08^b^	3.99 ± 0.11	4.50 ± 0.10^a^	3.68 ± 0.09^c^	4.36 ± 0.13^d,e^
Ct.Ar (mm^2^)	6.06 ± 0.06	5.88 ± 0.09	6.42 ± 0.11^b^	6.14 ± 0.08	5.76 ± 0.12^a^	6.53 ± 0.11^c^	6.03 ± 0.09^d,e^
Ct.Wi (mm)	0.64 ± 0.01	0.60 ± 0.01^a^	0.67 ± 0.01^b^	0.64 ± 0.01	0.58 ± 0.01^a^	0.69 ± 0.01^c^	0.61 ± 0.01^c,d,e^
Endocortical							
Ec.Pm (mm)	7.34 ± 0.09	7.78 ± 0.08^a^	7.38 ± 0.07^b^	7.52 ± 0.11	8.03 ± 0.11^a^	7.17 ± 0.11^c^	7.88 ± 0.10^d,e^
ES/BS (%)	23.50 ± 1.25	29.30 ± 1.49^a^	14.25 ± 1.96^b^	16.85 ± 1.74	31.76 ± 2.02^a^	14.81 ± 2.86^c^	37.51 ± 1.64^d,e^
Oc.S/BS (%)	4.36 ± 0.41	4.51 ± 0.43	1.73 ± 0.37^b^	2.35 ± 0.34	5.12 ± 0.70^a^	2.25 ± 0.68^c^	5.53 ± 0.74^d,e^
OS/BS (%)	2.06 ± 0.74	9.80 ± 1.64^a^	16.59 ± 1.95^b^	2.12 ± 0.95	15.65 ± 2.27^a^	0.00 ± 0.00^c,e^	6.77 ± 0.96^c,d,e^
MS/BS (%)	3.67 ± 0.81	8.46 ± 1.30^a^	26.59 ± 3.37^b^	4.59 ± 0.70	13.68 ± 1.89^a^	2.93 ± 0.61^c,e^	7.28 ± 0.92^c,e^
Periosteal							
Ps.Pm (mm)	11.55 ± 0.09	11.77 ± 0.08	11.82 ± 0.08	11.73 ± 0.10	11.76 ± 0.14	11.75 ± 0.12	11.85 ± 0.07
MS/BS (%)	35.96 ± 3.27	50.93 ± 3.85^a^	50.06 ± 2.11	31.36 ± 5.99	44.30 ± 2.72	40.46 ± 1.68^e^	41.71 ± 1.94^e^

Mean ± SEM (*n* = 10 for each group)

^a^ *P* < 0.05 versus Sham by unpaired *t* test

^b^ *P* < 0.05 versus Vehicle by unpaired *t* test

^c^ *P* < 0.05 versus Veh-Veh;

^d^ *P* < 0.05 versus PTH-IBN by Tukey’s multiple comparison test

^e^ *P* < 0.05 PTH versus PTH-IBN or PTH-Veh by Dunnett’s multiple comparison test

*Veh* vehicle, *IBN* ibandronate

PTH significantly decreased ES/BS and Oc.S/BS, and increased OS/BS and MS/BS in the endocortical femoral diaphysis (Table 3). In the PTH-IBN group, ES/BS, Oc.S/BS, OS/BS, and MS/BS in the endocortical femoral diaphysis were significantly lower than in the Veh-Veh group. In the PTH-Veh group, endocortical ES/BS and Oc.S/BS were not significantly different from those in the Veh-Veh group, and endocortical OS/BS and MS/BS were significantly lower than in the Veh-Veh group. Endocortical ES/BS and Oc.S/BS in the PTH-Veh group were significantly higher than in the PTH group, whereas in the PTH-IBN group they were not significantly different from those in the PTH group.

### Bone Turnover Markers

**Fig. 4:**
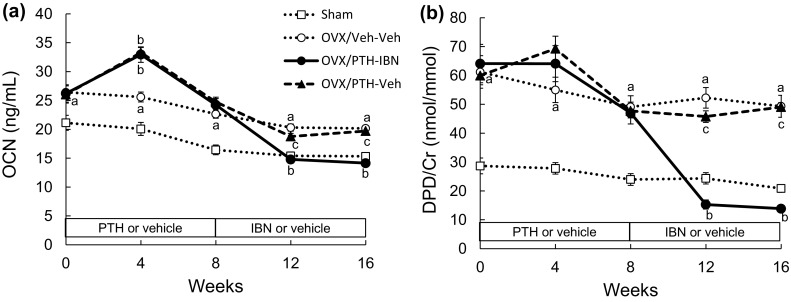
Time course of bone turnover markers. PTH(1–34) (10 µg/kg, s.c., five times/week) or vehicle was administered to ovariectomized (OVX) rats for 8 weeks from 8 weeks after OVX surgery. Ibandronate (10 µg/kg, s.c., every 4 weeks; IBN) or vehicle was administered sequentially for another 8 weeks following PTH treatment. Serum osteocalcin (**a** OCN) and urinary deoxypyridinoline normalized to urinary creatinine (**b** DPD/Cr) were measured every 4 weeks. Data are presented as mean ± SEM (*n* = 10 for each group). *a P* < 0.05 versus Sham by unpaired *t* test. *b P* < 0.05 versus Veh-Veh; *c P* < 0.05 versus PTH-IBN by Tukey’s multiple comparison test among OVX groups followed by Bonferroni correction

Serum OCN in the Veh-Veh group was significantly higher than that in the Sham group through the study period (Fig. 4a). PTH treatment significantly increased OCN at 4 weeks compared to the Veh-Veh group, and then OCN returned to the level of the Veh-Veh group at 8 weeks. Switching to IBN from PTH significantly decreased OCN compared to the Veh-Veh group, whereas switching to vehicle maintained OCN at the level of the Veh-Veh group.

Urinary DPD/Cr was significantly higher in the Veh-Veh group than in the Sham group (Fig. 4b). PTH treatment tended to increase DPD/Cr at 4 weeks, but not significantly. Switching to IBN significantly lowered DPD/Cr compared to the Veh-Veh group, whereas switching to vehicle did not change DPD/Cr.

## Discussion

The increase in BMD induced by PTH is rapidly lost after withdrawal of PTH [8–10]. It was reported that the gains in spinal areal BMD as well as spinal volumetric BMD were significantly lost at 12 months after completing 12-month therapy with PTH in postmenopausal osteoporotic women [9]. It was also reported that spinal BMD and biomechanical strength declined to the baseline levels at 8 weeks after withdrawal of PTH following 8-week treatment with PTH in OVX rats [21]. In the present study, PTH increased the BMD of the lumbar spine and the femur, and discontinuation of PTH decreased that increased BMD. We also observed a reduction in the biomechanical strength of the lumbar vertebra and femoral shaft after PTH withdrawal.

Bone microstructure as well as BMD contributes to bone biomechanical strength. Shahnazari et al. reported that 4-month withdrawal following 4-month treatment with PTH decreased the increased BV/TV and Tb.Th in the lumbar vertebrae and the increased cortical thickness in the femur in OVX rats [22]. In this study, 8-week discontinuation of PTH decreased BV/TV, which was increased by PTH, accompanied by a reduction in Tb.Th in trabecular bone of the L3 lumbar vertebra, and also decreased Ct.Ar and Ct.Wi, which were increased by PTH, accompanied by increased Ma.Ar in the cortical bone of the femoral shaft. This deterioration of trabecular and cortical architecture and the decrease in BMD resulted in a reduction in bone biomechanical strength after PTH withdrawal. The bone formation that is elevated by PTH treatment also decreases after discontinuation of PTH [9, 23]. In this study, histomorphometric analysis of the trabecular bone demonstrated that bone formation, which was increased by PTH, decreased after PTH withdrawal as indicated by significant decreases in OS/BS, Ob.S/BS, MS/BS, MAR, and BFR/BS in the PTH-Veh group as compared to in the PTH group.

To maintain the BMD and bone biomechanical strength increased by PTH, sequential therapy after PTH withdrawal is required. In this study, IBN was administered sequentially after PTH treatment in OVX rats. The BMD of the lumbar spine and the femur was lower in the PTH-Veh group than in the PTH group, whereas the BMD in the PTH-IBN group was not significantly different from that in the PTH group and was higher than in the PTH-Veh group. These results indicated that switching to IBN from PTH maintained the BMD increased by PTH at the level it was before switching. Switching to IBN also maintained the biomechanical strength of the lumbar vertebrae at the level before switching. The values of the biomechanical parameters in the PTH-IBN group were greater than those in the PTH-Veh group, indicating that sequential treatment with IBN after PTH is effective with respect to the biomechanical strength of the lumbar vertebrae.

In terms of histomorphometry, switching to IBN maintained BV/TV at the level it was before switching, accompanied with maintained Tb.Th. IBN also prevented the OVX-induced decrease in Tb.N, although PTH did not alter Tb.N. IBN resulted in a decrease in bone turnover after PTH treatment as shown by the decreases in both the bone resorption and bone formation parameters in the PTH-IBN group. These results suggested that switching to IBN decreases bone turnover and preserved the microstructure of the trabecular bone, which could contribute to maintaining the biomechanical strength of the lumbar vertebrae after withdrawal of PTH.

PTH improved the biomechanical strength of the femoral shaft. Maximum load and work to failure of the femoral shaft were lower in the PTH-Veh group than in the PTH group, whereas these parameters in the PTH-IBN group showed no significant difference compared to the PTH group and were higher than in PTH-Veh group indicating that IBN maintained the biomechanical strength of the cortical bone after PTH withdrawal. Histomorphometric analysis of the femoral diaphysis revealed that PTH increased cortical bone accretion at the endocortical surface as shown by the increases in Ct.Ar, Ct.Wi, and endocortical MS/BS and the decreases in Ma.Ar and Ec.Pm. Switching to IBN maintained the microstructure of the cortical bone by reducing bone turnover as indicated by the maintenance of Ct.Ar, Ct.Wi, Ma.Ar, and Ec.Pm at the levels of the PTH group, and the decreases in endocortical OS/BS and MS/BS as compared with the PTH group. These results suggested that the maintenance of the biomechanical strength of the femoral shaft by switching to IBN can be attributed to IBN’s effects on cortical microstructure as well as on BMD.

The concern with PTH therapy in osteoporosis is that the use of PTH is limited to no more than 2 years and that treatment with anti-resorptive agents sequentially after withdrawal of PTH is required to maintain bone mass. Daily treatment with alendronate immediately after the discontinuation of PTH maintains or increases BMD of the lumbar spine and femoral neck in postmenopausal women with osteoporosis [9, 12]. Weekly treatment with zoledronate after PTH withdrawal also inhibits the decrease in bone mass in OVX rats [24]. IBN is administered monthly in Japan for the treatment of osteoporosis [14]. In the present study, we administered IBN once every 4 weeks to OVX rats, and demonstrated that this long-interval dosing regimen of IBN could maintain bone mass and bone biomechanical strength after PTH withdrawal. IBN maintained BMD, biomechanical strength, and microstructure not only of the trabecular bone but also of the cortical bone after PTH withdrawal. The affinity of bisphosphonates for bone mineral influences their distribution within bone, and it is suggested that bisphosphonates with a lower affinity for bone mineral can penetrate more deeply into cortical bone [25]. IBN has comparatively low mineral-binding affinity; therefore, this lower affinity may contribute to the beneficial effect of IBN on cortical bone [26].

In addition to increasing cortical thickness or Ct.Ar, PTH treatment has also been reported to increase cortical porosity in monkeys as well as humans [27, 28]. However, in rats cortical porosity by intermittent administration of PTH is not observed due to the lack of intracortical remodeling, and in this study we found no cortical porosity after PTH administration in the femoral midshaft by histological observation (data not shown). Since Iwamoto et al. have demonstrated that ibandronate decreased PTH-induced cortical porosity in the femoral diaphysis in rabbits [29], it is possible that when significant intracortical remodeling is present in animal models as it is in human, ibandronate may reduce cortical porosity induced by PTH.

In summary, we investigated the effects of intermittent IBN treatment after withdrawal of PTH in OVX rats. IBN suppressed bone turnover and maintained BMD, biomechanical strength, and bone microstructure in the trabecular bone of the lumbar spine and in the cortical bone of the femur. It is expected that these results will be confirmed in patients with osteoporosis treated with IBN after withdrawal of PTH.
